# Pathogen-free, plasma-poor platelet lysate and expansion of human mesenchymal stem cells

**DOI:** 10.1186/1479-5876-12-28

**Published:** 2014-01-27

**Authors:** Paola Iudicone, Daniela Fioravanti, Giuseppina Bonanno, Michelina Miceli, Claudio Lavorino, Pierangela Totta, Luigi Frati, Marianna Nuti, Luca Pierelli

**Affiliations:** 1 Immunohematology and Transfusion Medicine, San Camillo Forlanini Hospital, Rome, Italy; 2 Department of Gynecology, Catholic University Medical School, Rome, Italy; 3 Department of Experimental Medicine, Sapienza University, Rome, Italy; 4 Department of Science, Section Biomedical Science and Technology, University “Roma Tre”, Rome, Italy

**Keywords:** Blood component preparations, Cellular therapy, Mesenchymal stem cells, Platelet lysate

## Abstract

**Background:**

Supplements to support clinical-grade cultures of mesenchymal stem cells (MSC) are required to promote growth and expansion of these cells. Platelet lysate (PL) is a human blood component which may replace animal serum in MSC cultures being rich in various growth factors. Here, we describe a plasma poor pathogen-free platelet lysate obtained by pooling 12 platelet (PLT) units, to produce a standardized and safe supplement for clinical-grade expansion of MSC.

**Methods:**

PL lots were obtained by combining 2 6-unit PLT pools in additive solution (AS) following a transfusional-based procedure including pathogen inactivation (PI) by Intercept technology and 3 cycles of freezing/thawing, followed by membrane removal. Three PI-PL and 3 control PL lots were produced to compare their ability to sustain bone marrow derived MSC selection and expansion. Moreover, two further PL, subjected to PI or not, were also produced starting from the same initial PLT pools to evaluate the impact of PI on growth factor concentration and capacity to sustain cell growth. Additional PI-PL lots were used for comparison with fetal bovine serum (FBS) on MSC expansion. Immunoregulatory properties of PI-PL-generated MSC were documented *in vitro* by mixed lymphocyte culture (MLC) and peripheral blood mononuclear cells (PBMC) mitogen induced proliferation.

**Results:**

PI-PL and PL control lots had similar concentrations of 4 well-described growth factors endowed with MSC stimulating ability. Initial growth and MSC expansion by PI-PL and PL controls were comparable either using different MSC populations or in head to head experiments. Moreover, PI-PL and PL control sustained similar MSC growth of frozen/thawed MSC. Multilineage differentiation of PI-derived and PI-PL-derived MSC were maintained in any MSC cultures as well as their immunoregulatory properties. Finally, no direct impact of PI on growth factor concentration and MSC growth support was observed, whereas the capacity of FBS to sustain MSC expansion in basic medium was irrelevant as compared to PL and PI-PL.

**Conclusion:**

The replacement of animal additives with human supplements is a basic issue in MSC *ex vivo* production. PI-PL represents a standardized, plasma-poor, human preparation which appears as a safe and good candidate to stimulate MSC growth in clinical-scale cultures.

## Background

Mesenchymal stem cells (MSC) are multipotent progenitors cells with self-replicative and differentiation abilities. Actually, MSC differentiate into several tissues of mesoderm lineage, although their potential to give rise to cells of several embryonic origin as neurons, hepatocytes or epithelial cells has also been reported [1-3]. With their plasticity, self-renewal and immunoregulatory capacities these cells have generated great interest for clinical applications either in cell therapy aimed to induce immune tolerance or in tissue engineering [4-11].

MSC are typically obtained from bone marrow (BM), but they can be also isolated from a variety of other human sources as adipose tissue, muscle, connective tissue, dental pulp, cord blood, placenta and amniotic fluid [12,13]. In BM, MSC are present at a very low frequency (0.001-0.01%), thus *ex-vivo* expansion is an essential step to reach a number of MSC which appears appropriate for clinical applications. To sustain cell growth, most clinical-scale MSC production protocols use cocktails which contain serum of animal origin as supplement*.* However, these products maintain the potential risk of pathogen transmission and immunological reactions related to the different species origin. Platelet lysate (PL) contains a wide series of growth factors, thanks to which platelets (PLT) are capable to mediate tissue repair at injured sites in physio-pathological conditions; for these reasons it has been proposed as a potential supplement for MSC cultures. Various studies have demonstrated that growth factors derived from PL are able to sustain MSC growth and expansion [14-18] and, since these observations have been reported, several efforts have been made to standardize its production. In regard to this issue, it has to be taken into account that the concentration of soluble growth factors released by PLT is highly variable among different individuals. Therefore, PLT from multiple donors should be required and included in each preparation to compensate individual variability and to obtain a more standardized and reproducible PL product. Pooling PLT obtained by whole blood-derived buffy-coats is a standardized procedure to produce pooled PLT concentrates for transfusional use. Considering that transmission of pathogens via blood transfusion is still a major threat, plasma or PLT pathogen inactivation (PI) has been introduced for routine blood component production at several sites*.* The innovative technology of photochemical PI utilizes a synthetic psoralen, as active compound, which specifically interacts with nucleic acids when exposed to UVA light, blocking both DNA or RNA replication. Thus, the technology shows efficacy in inactivating viruses, bacteria, protozoa and eventual residual leucocytes. Starting from these concepts, we adopted a PLT pooling procedure followed by an additional step of photochemical treatment necessary for PI to produce a plasma-poor, pathogen-free PL in a closed sterile system. The pathogen inactivated PL (PI-PL) preparations were employed to sustain the growth and the expansion of MSC from different BM samples. This preparation was named Mesengen™ by a trademark associated with the registration of the international patent application of this product (PCT/IB2012/055062).

## Methods

### Protocol for clinical-scale preparation of plasma-poor PI-PL

**Figure 1:**
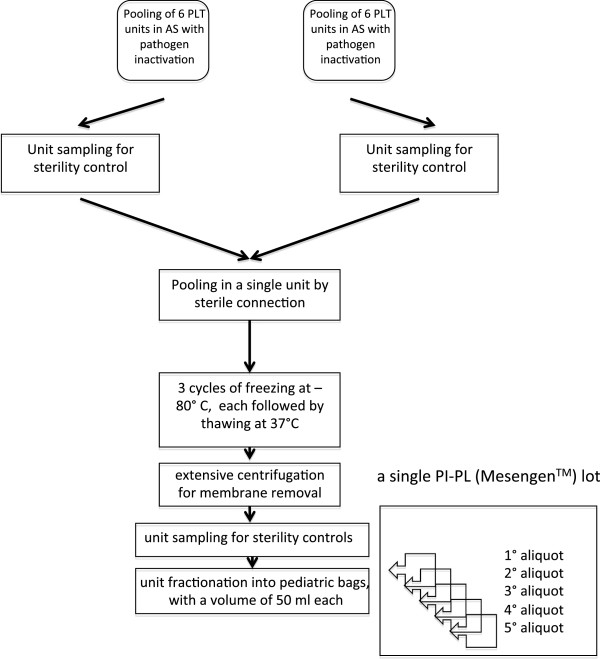
**PI-PL production process flow-chart.** The scheme shows the production process of a single PI-PL lot starting from two pools of 6 PLT units each. PLT, platelets; AS, additive solution.

Whole blood was collected from voluntary donors selected following current procedures for blood donation. Blood units were screened for transfusion transmitted viruses and other blood-borne pathogens in compliance with national regulatory requirements. Buffy-coats (BC) were obtained by centrifugation of whole blood donations according to the procedures validated in the routine separation of blood components for transfusional therapy. Six BC-PLT units were pooled within 24 hours from collection by using a sterile connector device (SCD - Composeal, Fresenius Kabi AG, Bad Homburg, Germany) and a dedicated set for component pooling and production (Terumo-Teruflex BP-kit with Imugard III-S-PL, Terumo, Tokyo, Japan). After centrifugation at 1,300 rpm for 12 minutes at 22°C (Heraeus Cryofuge 5500i, Thermo Fisher Scientific, Waltham, MA, USA) PLT were separated from BC by using the automatic system Compomat G4 (Fresenius Kabi AG). The pool of six PLT units underwent PI procedure by the Intercept technology (Intercept Blood System for Platelets, Cerus Corporation, Concord, California, USA). The entire process was performed in a closed system and based on the following major steps: a) mixing of PLT pool with psoralen compound (Amatosalen; Cerus Corporation), b) illumination with UVA light, c) removal of residual psoralen and free photoproducts by means of a compound absorption device (CAD). At the end of the process, the inactivated PLT were resuspended in InterSol solution (additive solution, AS; Fenwall Inc., Lake Zurich, IL, USA) containing a residual 20-30% of human plasma (for a total volume of approximately 300 mL), with a PLT content ranging from 2.5 to 3.5 × 10^11^. The clinical-scale process to generate a single lot of a PI-PL is summarized in Figure 1. In detail, two pools of six PI-PLT units were connected each other under sterile conditions to obtain a pool of 12 PI-PLT units, provided that they had a PLT count equal to 1 × 10^9^/mL or slightly higher. The final pooled-PLT product was frozen at -80°C and thawed at 37°C for three consecutive cycles to lyse PLT, then centrifuged at 4,000 rpm for 30 minutes at room temperature (Heraeus Cryofuge 6000i, Thermo Fisher Scientific) to remove PLT membranes. Sterility tests were performed prior and after the entire procedure of PL production to exclude aerobic, anaerobic and fungal contamination (BacT/ALERT, Biomerieux SA, Marcy l’Etoile, France). The PI-PL preparation was finally collected into pediatric bags (50 mL per bag) and cryopreserved at -80°C until use.

Control PL preparations were also produced for comparison following the same procedure above described without including the PI step.

On the whole, 11 PL preparations were tested and shown in the present work: three inactivated (PI-PL1, PI-PL2, PI-PL3), three as controls (PL1, PL2, PL3), one aliquoted in two parallel samples prior PI to obtain a PL and a PI-PL from the same lot (PL18 and PI-PL18) and three additional PI-PL to make a comparison with fetal bovine serum (FBS) on MSC growth (PI-PL11, PI-PL16, PI-PL43/44). Preparations were tested to compare the ability of both PL and PI-PL products to sustain growth and expansion of MSC isolated from BM obtained from 14 different donors.

### Measurements of PLT derived growth factors in PI-PL and control PL preparations

The concentration of PLT growth factors, *i.e.* platelet derived growth factor AB (PDGF-AB), vascular endothelial growth factor (VEGF), basic-fibroblast growth factor (bFGF) and transforming growth factor-β1 (TGF-β1, in the immunoreactive form) was evaluated in triplicate in each PI-PL and control PL preparation by using commercially available immunoenzymatic kits: PDGF-AB (Quantikine Human PDGF-AB, R&D Systems, Minneapolis, Minnesota, USA), VEGF (Quantikine Human VEGF, R&D System), bFGF (Quantikine Human FGF basic, R&D System), TGF-Beta (Quantikine Human TGF-Beta 1, R&D System).

### MSC selection from BM and in-vitro expansion

BM derived mononuclear cells (BM-MNC) were obtained by wash-out of BM left over contained in bags and filters following BM manipulation for clinical transplantation [19]. Fourteen different BM samples were obtained from as many donors of BM grafts, following a clinical-scale procedure with GMP-grade reagents. BM left over contained in bag and filters was washed with sterile PBS GMP-grade (LiStarFish, Milano, Italy) and BM-MNC were isolated by density gradient (lymphocyte separation medium 1,077 GMP-grade, LiStarFish). BM-MNC were washed twice in PBS GMP-grade additioned with 2% human albumin (Kedrion S.P.A Lucca, Italy), counted and suspended at 1 × 10^6^ BM-MNC/mL in culture medium consisting of D-MEM GMP-grade (LiStarFish) additioned with 10% PI-PL or 10% PL control. Moreover, in some experiments BM-MNC were cultured in D-MEM additioned with 10% FBS (EuroClone, Milan, Italy). The cells were cultured in T25 or T75 polystyrene flasks (Iwaki, Asahi Glass Co LTD, Tokyo, Japan) and maintained at 37°C in a humidified atmosphere containing 5% CO_2_. After 48 hours the non-adherent cells were removed and fresh medium added to the cultures. The medium was then changed twice a week until cells reached a confluence degree of 80% (passage 1, P1), as judged by microscopical examination at 10× and 4× magnification. At confluence, cells were detached by trypsin (GMP-grade, Li StarFish) treatment, harvested, counted and diluted to 4,000 cells/cm^2^ for further expansion. In order to avoid the expected inaccurancy of cell counting by a manual method, MSC enumeration during cultures was performed by flow cytometry and expressed as absolute count, using monoclonal antibodies (Mo-Ab) specific for a constitutive antigen of MSC (CD105) and an additional Mo-Ab, with CD90 or CD44 specificity, which co-identifies MSC. Briefly, pre-defined amounts of fluorescent beads were added to a known volume of cells and counted along with cells by using a FacsCalibur (Becton-Dickinson San Jose’, California, USA). Cells were gated on the basis of their physical features (forward and side scatter signals) and fluorescent signals. Two replicates counts were performed for each sample and the average value of replicates was accepted as the correct absolute cell number.

Once MSC reached confluence in each respective culture (P1), nascent MSC populations were identified as MSC-1, MSC-2, MSC-3, MSC-4, MSC-5, MSC-6, MSC-7, MSC-8, MSC-9, MSC-11, MSC-12, MSC-082, MSC-170 and MSC-180. Following P1, MSC underwent two further passages (passage 2 and 3, P2 and P3) and fold expansion was calculated by dividing the total number of cells obtained at confluence by the number of cells seeded at each passage. At the end of expansion (P3), aliquots of 2 × 10^6^ MSC were frozen in D-MEM additioned with 5% human albumin and 10% dymethyl-sulfoxide (DMSO) GMP-grade (LiStarFish) as final concentration, by using a freezing protocol (Kryo 560-16 Planer; Planer PLC Sunbury-On-Thames, United Kingdom) validated for cryopreservation of haemopoietic stem cells for clinical transplantation. MSC were cryopreserved in liquid nitrogen for at least 3 months, then thawed, washed and checked for viability. After thawing, 15,000 viable MSC/cm^2^ were cultured again in D-MEM additioned with the same PL or PI-PL preparations used for their pre-freezing expansion. At 80% confluence cells were detached by trypsin treatment, analyzed by flow–cytometry following the previously described analytical strategy and re-expanded to evaluate the ability of MSC to proliferate and differentiate after cryopreservation from passage 3 to 4 (P3, P4).

### Colony-forming unit-fibroblast (CFU-F) assay

Colony-forming-unit assay was performed by plating 2 × 10^5^ BM-MNC/ml in D-MEM medium additioned with 10% of PL or 10% of PI-PL. Clusters consisting of at least 50 cells were counted as a colony under an inverted microscope.

### MSC immunophenotyping

Flow cytometric analysis was performed on starting samples and at each passage by using the following Mo-Abs: anti-CD71 (allophycocyanin, APC-conjugated, Becton-Dickinson), anti-CD73 (phycoerytrhin, PE-conjugated, Becton-Dickinson), anti-CD90 (allophycocyanin, APC-conjugated, Becton-Dickinson), anti-CD105 (peridinin chlorophyll protein complex, PerCP-conjugated, Becton-Dickinson), anti-CD166 (PE conjugated, Becton-Dickinson), anti-CD45 (FITC-conjugated, Becton-Dickinson) and anti-CD34 (FITC-conjugated, Becton-Dickinson).

At the end of the culture time, MSC were checked for viability by using (7-amino-actinomycin D) 7-AAD (Becton-Dickinson) and a Mo-Ab identifying CD105, which represents a MSC constitutive antigen. Viable MSC were identified as CD45-, CD105+, 7-AAD- cell events.

### MSC immunoregulatory effect

**Figure 2:**
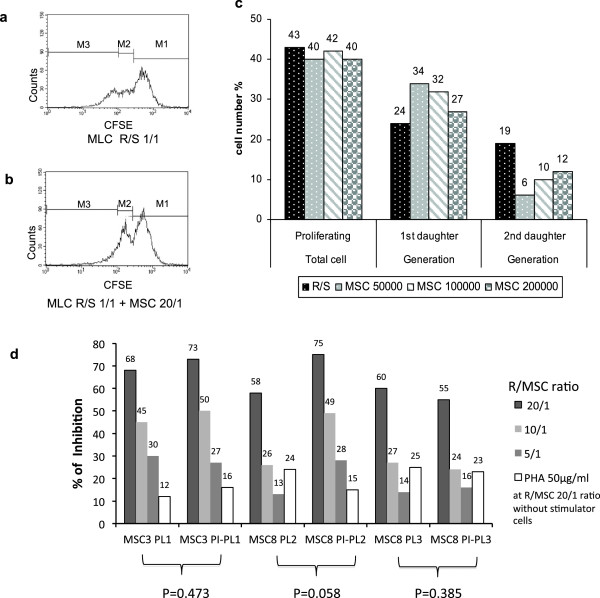
**Experiments of MSC induced immunosuppressive activity.** CSFE dilution profile of stained responder (R) cells in MLC cultured without MSC **(a)** and with MSC, with R cells to MSC ratio of 20/1 (R/MSC 20/1) **(b)**. M1 is the peak of cells not proliferating, M2 is the peak of 1st daughter proliferating cells and M3 is the peak of 2nd daughter proliferating cells. **c)** Percentage of R proliferating cells in MLC moving respectively to 1st and 2nd daughter generations either in absence or in presence of different MSC concentrations, in a representative experiment. **d)** Inhibition of cell proliferation in MLC expressed as percentage of study R cells (co-cultured with MSC) not moving from 1st daughter to 2nd daughter generation, compared to control R cells (cultured without MSC; see also methods). The experiments were performed using PL1- and PI-PL1derived MSC3, PL2- and PI-PL2-derived MSC8, PL3- and PI-PL3-derived MSC8. PBMC used as R cells in MLC were also co-cultured at R/MSC 20/1 without stimulator cells with PHA at a concentration of 50 μg/ml. Data show that with a ratio of R cells to MSC of 20/1 (R/MSC 20/1) the highest cell proliferation inhibition was observed, without any significant difference among MSC populations (as judged by two-tailed paired Student’s T test, specifically applied to assess differences between PL- and PI-PL-derived identical MSC populations). Cultures carried out in the presence of PHA produced less inhibition for any MSC population than the corresponding MLC at R/MSC 20/1. MSC, mesenchymal stem cells; R, responder cells; S, stimulator cells; MSC 50000 corresponds to R/MSC 20/1, MSC 100000 to R/MSC 10/1, MSC 200000 to R/MSC 5/1.

Peripheral blood mononuclear cells (PBMC), from different voluntary donors after informed consent, were isolated by density gradient and used as responder (R) and stimulator (S) cells in Mixed Lymphocyte Cultures (MLC) or in mitogen proliferation assay by phytohaemagglutinin (PHA; Gibco, Life Technologies Italia, Monza, Italy). MSC were added to MLC and PHA-stimulated lymphocyte culture and the proliferation was evaluted by flow-cytometric analysis of the cell tracking dye carboxy-fluorescein di-acetate succinimidyl ester (CFSE; Invitrogen, Life Technologies). Briefly, 5 × 10^6^ R cells were stained with CFSE 5 μM and then checked for viability. 1 × 10^6^ viable CFSE+ R cells were cultured with 1 × 10^6^ irradiated S cells at 1/1 ratio in MLC or with 50 μg/mL of PHA, respectively for 7 and 3 days at 37°C in a humidified atmosphere containing 5% CO_2_. At beginning of the cultures, third-party MSC, derived from PL or PI-PL driven expansion process, were added at a R cells/MSC ratio of 20/1, 10/1, 5/1. Following antigen or mitogen proliferative stimulus, the stained cells undergo mitotic events which generate a CFSE dilution profile. Generational boundaries identify adjacent daughter peaks which are used to calculate percentage of proliferating cells falling in each daughter peak. The inhibition of cell proliferation in MLC, as well as in PHA cultures, was expressed as the percentage of study R cells (co-cultured with MSC) not moving from 1st daughter (peak M2, see Figure 2) to 2nd daughter generation (peak M3), compared to control R cells (cultured in the absence of MSC) and was calculated by the following formula:

$$\%\;\mathrm{Inhibition}=1‒\frac{\%\;\mathrm{study}\;R\;\mathrm{cells}\;\mathrm{in}\;M3}{\%\mathrm{control}\;R\;\mathrm{cells}\;\mathrm{in}\;M3}\times 100$$

Experiments were performed using PL1- or PI-PL1-derived MSC3, PL2- ,PI-PL2- ,PL3- and PI-PL3-derived MSC8 (Figure 2).

**Figure 3:**
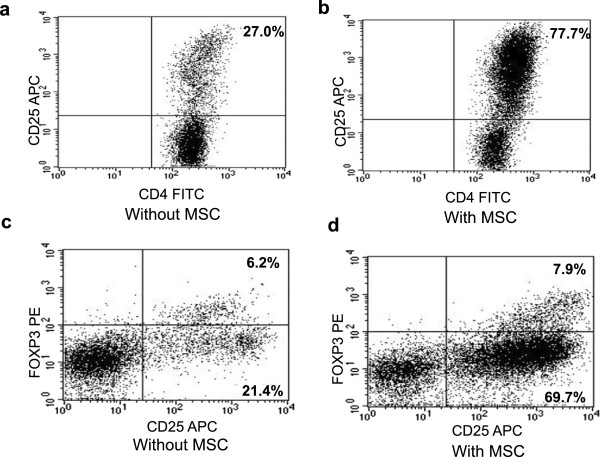
**Generation of regulatory T cells by MSC.** Expression of CD4 + CD25+ and CD25 + FoxP3+ in PBMC 14-day cultured without **(a, c)** or with PI-PL3-derived MSC8 **(b, d)** at a ratio PBMC/MSC 5/1, showing nascent regulatory T cells at higher frequency in the presence of MSC.

PBMC and MSC were also co-cultured at a ratio of 5/1 for 14 days and, at the end of culture, stained with antibodies against CD4 (FITC-conjugated, Becton-Dickinson), CD25 (APC-conjugated, Becton-Dickinson) and FoxP3 (PE conjugated, Becton-Dickinson) to evaluate the ability of MSC to stimulate the proliferation of T regulatory CD4 + CD25 + FoxP3+ cells (Figure 3) [20].

### MSC differentiation assays

The potential of MSC to differentiate into different mesodermal lineages was assessed at P3 according to the following protocols:

*Osteogenesis* - cells were cultured at 20,000 cell/cm^2^ in Petri dishes in α-MEM (Invitrogen, Life Technologies) supplemented with 5% of control PL or 5% of PI-PL, desamethasone 10^-7^ M (Sigma-Aldrich, St Louis, Missouri, USA), ascorbic acid 10^-5^ M (Sigma-Aldrich), β-glycerophosphate 10^-2^ M (Sigma-Aldrich). MSC unexposed to differentiating factors, were cultured in α-MEM additioned with either 5% of PL or PI-PL and used as staining control. After 21 days of culture the cells were stained with naftol AS-O cloride-acetate and α-naftil acetate esterase (Sigma-Aldrich) and subjected to examination by an optical microscope with a magnification of 10× and 40×.

*Adipogenesis* - cells were cultured at 20,000 cell/cm^2^ in Petri dishes in α-MEM (Invitrogen, Life Technologies) supplemented with 5% of control PL or 5% PI-Pl, desamethasone 10^-6^ M (Sigma-Aldrich), insulin 10 μg/mL (Sigma-Aldrich), 1-methyl-3-isobutylxhantine 5 × 10^-4^ M (Sigma-Aldrich), indomethacin 60 μM (Sigma-Aldrich). Unstimulated MSC cultured in α-MEM additioned either with 5% of PL or PI-PL were used as staining control. After 21 days of culture the cells were stained with oil RED O (Bio-Optica, Milano, Italy) and evaluated by microscopic examination.

*Chondrogenesis* - 200,000 cells were cultured in 15 mL conical tube in α-MEM (Invitrogen, Life Technologies) supplemented with 5% of control PL or 5% PI-PL, desamethasone 10^-7^ M (Sigma-Aldrich), ascorbic acid 10^-4^ M (Sigma-Aldrich), transforming growth factor-beta 10 ng/mL (Sigma-Aldrich) and insulin 10 μg/mL (Sigma-Aldrich). After 21 days of culture, cells were harvested, fixed with formalin and embedded with paraffin. Sections were stained with toluidine blue (Bio-Optica, Milano, Italy) and evaluated by microscopic examination. 200,000 unstimulated MSC were cultured in Petri dishes in α-MEM additioned either with 5% of PL or PI-PL and used as staining control.

### Karyotypic and statistical analysis

All MSC populations, when reached P1 and P3 (P4 for frozen/thawed MSC), were cultured at a cell concentration of 7,000 cells/cm^2^ in identical culture conditions as those described above to evaluate karyotypic analysis according to analytical protocols of the International System for Human Cytogenetics Nomenclature.

### Statistical analysis

Data obtained from growth factor assays, cell growth experiments, flow cytometry and functional studies were compared using two-tailed unpaired, two-tailed paired Student’s T test, ANOVA and Chi-square for multiple dependent variables, as appropriate (StatPlus:mac, AnalystSoft Inc., Alexandria, Va, USA). A p value < 0.05 was considered as statistically significant.

## Results and discussion

### Control PL and PI-PL preparations

Six PL preparations were produced by the clinical-scale procedure, three inactivated (PI-PL1, PI-PL2, PI-PL3) and three as controls (PL1, PL2, PL3). Additional PL and PI-PL were then produced by identical procedures and tested to assess the direct impact of PI on growth factors and growth-promoting ability (PL18, PI-PL18) and to compare FBS and PI-PL (PI-PL11, PI-PL16, PI-PL43/44) effect on MSC growth. All preparations gave negative cultures at the sterility controls and therefore were used for MSC selection and expansion protocols.

The concentration of PDGF-AB, VEGF, bFGF and TGF-β1 released in PL were comparable between PL control and PI-PL lots and among the different preparations, suggesting that pooling of 12 PLT units in a single unit may balance the individual variability of PLT growth factor release into the final product (Table 1).

**Table 1:** Analysis of growth factor concentration by ELISA in different PL and PI-PL preparations

**PL preparations**	PDGF-AB* (pg/ml)	**VEGF**^**§**^** (pg/ml)**	**bFGF° (pg/ml)**	**TGF-beta1**^**# **^**(pg/ml)**
**PL1**	42,778 ± 10,206	15,666 ± 1,257	94.6 ± 6.11	88,590 ± 3,169
**PL2**	32,071 ± 2,525	11,239 ± 673	111.6 ± 1.40	72,694 ± 3,245
**PL3**	26,272 ± 1,105	10,571 ± 514	91.3 ± 3.21	90,637 ± 533
**PI-PL1**	25,586 ± 1,281	11,276 ± 1,108	110 ± 10	63,512 ± 1,500
**PI-PL2**	35,140 ± 1,624	10,119 ± 827	118 ± 2.88	70,626 ± 2,013
**PI-PL3**	41,299 ± 2.041	11,187 ± 757	109 ± 3.60	90,188 ± 1.051

*PL* platelet lysate, *PI-PL* pathogen inactivated platelet lysate, *PDGF-AB* platelet derived growth factor AB, *VEGF* vascular endothelial growth factor, *bFGF* basic fibroblast growth factor, *TGF-beta1* transforming growth factor beta1; means ± standard deviations are shown of a triplicate assay performed to detect each growth factor concentration. *p = 0.937, ^§^p = 0.119, °p = 0.009 (in favour of PI-PL), ^#^p = 0.082 at unpaired two-tailed Student’s T test between concentrations observed in control PL and PI-PL lots.

### Effect of control PL and PI-PL on MSC selection and expansion

**Figure 4:**
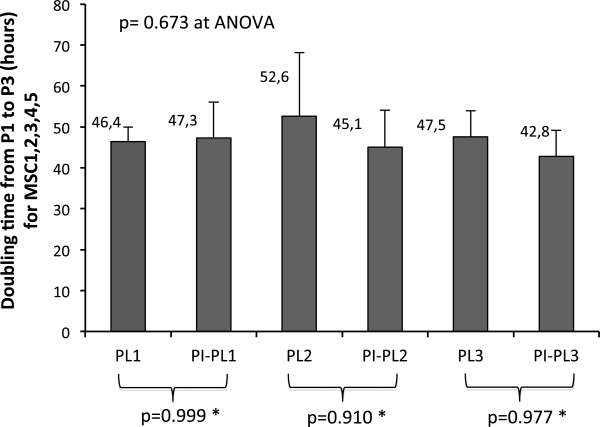
**MSC growth in presence of three different lots of PL and PI-PL.** MSC 1,2,3,4,5 were assessed in their growth ability by measuring doubling time (DT) for each culture experiment, in the presence of PL1,2,3 and PI-PL1,2,3 series. Data are presented as the average ± standard deviation of DT obtained in the presence of each PL or PI-PL for different MSC populations. Statistical comparison was made by ANOVA which states the absence of any significant difference among culture series. *Statistical significance was evaluted between the indicated groups by post-hoc Scheffe’s F test.

**Figure 5:**
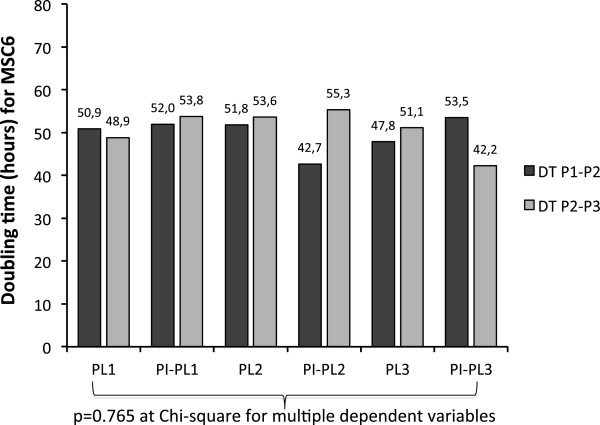
**Parallel growth of MSC6 population sustained by PL1, PL2, PL3 and PI-PL1, PI-PL2, PI-PL3 lots.** Data are expressed as doubling time (DT) from passage 1 to passage 2 (DT P1-P2) and from passage 2 to passage 3 (DT P2-P3). Coefficient of variation for PL1,2,3 and PI-PL1,2,3 series was 4.14% and 11%, respectively, during P1-P2 passage and 5% and 13%, respectively, during P2-P3 passage. No differences between groups were revealed by Chi-square for multiple dependent variables.

**Figure 6:**
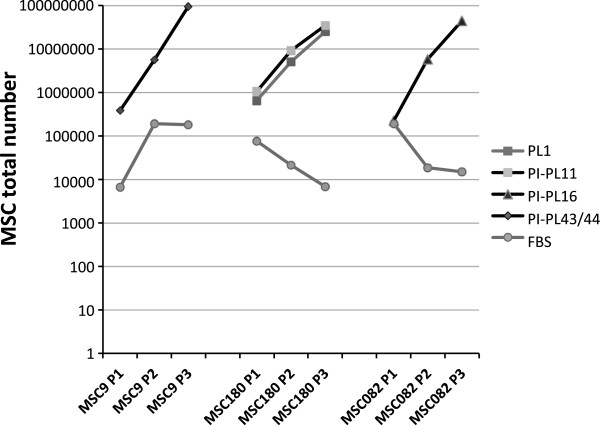
**MSC growth in the presence of fetal bovine serum (FBS) or PL/PI-PL lots.** Growth of three MSC populations (MSC9, MSC180, MSC082) at passage1,2,3 (P1,P2,P3) in the presence of PL1, PI-PL11, PI-PL16, PI-PL43/44 and FBS, expressed as the absolute number of cells obtained in cultures in D-MEM medium. Cell number, expressed by a logarithmic scale, shows in any case an irrelevant effect of FBS on MSC growth when used as a supplement of a basic culture medium.

By culturing 5 × 10^6^ MNC isolated from BM samples, a median of 0.931 × 10^6^ MSC (range 0.364 × 10^6^-1.264 × 10^6^) were obtained within 15 days (range 12-16 days) in all growth experiments, including all cultures carried out in the presence of both PL control and PI-PL lots. Figure 4 shows the average magnitude of five MSC (identified as 1,2,3,4,5) growth (from P1 to P3) expressed as doubling time (DT) which was comparable for PI-PL preparations and PL controls. Then, MSC6 sample was chosen to test three different PI-PL lots (1,2,3) in comparison with controls PL1, PL2 and PL3. Figure 5 shows as the different PI-PL preparations exerted a growth effect, expressed as DT for both P1-P2 and P2-P3 passages, comparable to PL1, PL2 and PL3 controls and in any case capable of inducing at least a 90 fold expansion (expressed as the expansion from P1 to P3 phase; data not shown). An additional experiment was carried out to test the ability of different PI-PL lots to promote the growth of MSC originating from the same initial BM-MNC suspension, in a head to head comparison with PL controls. The comparisons between the MSC growth by PL and PI-PL lots expressed as fold expansion and DT are reported in Table 2. Results of this set of growth experiments carried out on MSC1, MSC3, MSC4, MSC5, MSC6, MSC7 and MSC8 indicated that the process of inactivation does not compromise the capacity of each PI-PL lot to initiate MSC growth (P1) and to promote expansion at P3, with proper and comparable DT. On the whole, the results of MSC growth experiments show that both control PL and PI-PL preparations are able to exert a proper stimulus for MSC proliferation. On the contrary, FBS added to D-MEM without other additives produced an irrelevant effect on MSC culture initiation and subsequent expansion, as compared to four different PL/PI-PL lots (PL1, PI-PL11,PI-PL16,PI-PL43/44, Figure 6).

**Table 2:** Comparisons between control PL and PI-PL lots to support expansion of the same MSC populations

**MSC**	**PL or PI-PL**	**P1**		**P3**		Fold Expansion*	**Doubling Time (hours)°**
		**Day to reach**	**MSCx10**^**6**^^**§**^	**Day to reach**	**MSCx10**^**6**^^**#**^		
**MSC-1**	**PL1**	16	0.847	27	142	168	35.7
	**PI-PL1**	16	0.765	27	120	157	36.2
**MSC-3**	**PL1**	16	0.572	31	182	319	43.3
	**PI-PL2**	16	0.564	31	177	313	43.4
**MSC-6**	**PL1**	15	1.150	27	116	97	43.3
	**PI-PL3**	15	1.264	27	162	128	41.1
**MSC-3**	**PL2**	15	0.933	32	204	219	52.5
	**PI-PL1**	15	0.950	32	212	223	52.3
**MSC-4**	**PL2**	13	0.560	31	101	181	57.6
	**PI-PL2**	13	0.638	31	77	177	62.5
**MSC-7**	**PL2**	15	0.570	27	46	81	45.5
	**PI-PL3**	15	0.563	27	83	148	40
**MSC-5**	**PL3**	12	0.940	30	144	154	59.5
	**PI-PL1**	12	0.948	30	117	123	62.2
**MSC-6**	**PL3**	13	1.056	31	100	95	65.8
	**PI-PL2**	13	0.899	31	103	115	63.2
**MSC-8**	**PL3**	13	0.970	32	109	112	66.9
	**PI-PL 3**	13	1.155	32	113	98	68.9

*PL* platelet lysate, *PI-PL* pathogen inactivated platelet lysate, *MSC* mesenchymal stem cells, *P1* passage 1 of culture, *P3* passage 3 of culture; different MSC populations (1,3,4,5,6,7,8) were obtained by the culture of a starting number of 5 × 10^6^ mononuclear cells from as many bone marrow samples.

^§^p = 0.640 and ^#^p = 0.802 at paired two-tailed Student’s T test between MSC growth promoted by control PL and PI-PL lots. *fold expansion at P3 as compared to cells obtained at P1, p = 0.541 at paired two-tailed Student’s T test between MSC expansion promoted by control PL and PI-PL lots. °doubling time measured as hours between P1 and P3 for each MSC population, p = 0.975 at paired two-tailed Student’s T test between control PL and PI-PL lots.

**Figure 7:**
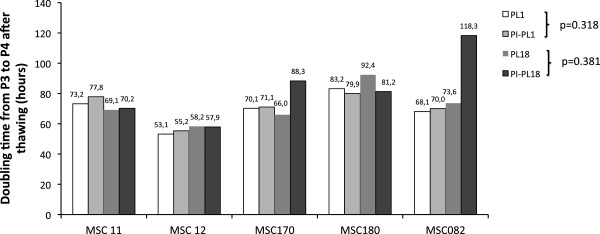
**Cryopreserved MCS expansion by Pl or PI-PL.** Re-expansion of cryopreserved MSC11,12,170,180 and 082 by control PL1, PL18 and PI-PL1, PI-PL18 expressed as doubling time (DT) observed between passage 3 (P3) and 4 (P4). Two-tailed paired Student’s T test performed between PL1 versus PI-PL1 and PL18 versus PI-PL18 lots shows no significant differences.

After thawing and washing to remove DMSO, MSC11 MSC12, MSC170, MSC180 and MSC082 showed a cell viability greater than 80% and the typical MSC surface phenotype. Thawed cells retained plastic adherence capacity, reaching confluence within 1 week from the start of culture. Results of MSC growth between passage 3 and 4 after thawing, expressed as DT, are shown in Figure 7. Both PI-PL and control PL preparations promoted comparable cell growth of different thawed MSC populations.

**Figure 8:**
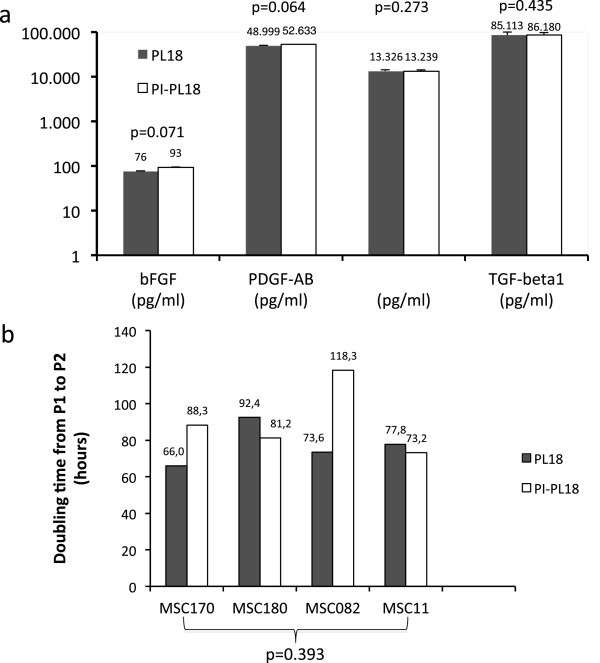
**Comparison of growth factor concentration and growth promoting-ability between PL18 and PI-PL18.** PL18 and PI-PL18 were generated by the same initial lot (omitting PI step in PL18 preparation) and were compared to assess the impact of PI on growth factors and MSC growth in a head to head comparison. **a)** growth factor concentrations were evaluated in triplicates for both samples and compared by two-tailed paired Student’s T test which shows no significant differences; **b)** MSC170, MSC180, MSC082 MSC11 populations were grown in parallel in the presence of either PL18 or PI-PL18; cell growth was expressed as doubling time (DT) between passage 1 (P1) and passage 2 (P2). Statistical analysis by two-tailed paired Student’s T test between PL18- or PI-PL18-induced growth on different MSC populations shows no signficance in the direct comparison.

The results of additional growth factor assays and MSC growth experiments carried out using PL18 and PI-PL18 (these two preparations were obtained from the same initial lot) are shown in Figure 8. These experiments, showing a direct comparison between PL18 and PI-PL18, confirm that PI did not exert any significant effect on both PDGF-AB, VEGF, bFGF and TGF-β1 concentrations and growth of MSC170, MSC180, MSC082 and MSC11.

Cultures were evaluated by karyotypic analysis at P3 (P4 for frozen/thawed MSC) and none abnormality had been revealed according to the analytical protocols of the International System for Human Cytogenetics Nomenclature (data not shown).

### CFU-F assays

The MSC precursor content was found comparable by culturing BM-MNC in medium additioned with PL or PI-PL. The number of CFU-F per million of BM-MNC was respectively 16 (PL) and 19 (PI-PL), on average.

### Flow cytometric analysis and differentiation properties of cultured MSC

**Figure 9:**
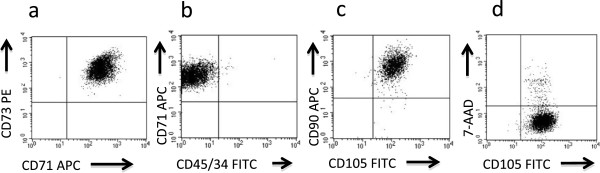
**Flow cytometric analysis of MSC6 cultured with PI-PL2.** A representative example of flow cytometric analysis of MSC6 population at P1 obtained from a culture established in the presence of PI-PL2. Dot plots **a)**, **b)**, and **c)** show co-expression of typical MSC markers on the majority of analysed cells with the absence of CD45 and CD34 haemopoietic cell markers. Dot plot **d)** shows a typical analysis to evaluate MSC viability by 7-AAD staining associated with the expression of CD105 constitutive antigen.

Flow cytometric analysis carried out on initial MNC from 14 BM samples showed that the MSC-associated phenotype, such as CD45^-^, CD34^-^, CD105^+^, CD90^+^, CD73^+^, CD71^+^, CD166^+^, was rarely expressed among these cells (<0.05%), whereas at P1 more than 95% of the cells homogeneously expressed the MSC phenotype, in cultures additioned with both control PL and PI-PL. Comparable expression of MSC surface antigens was observed in all cultures at different passages with a cell viability never lower than 90% at any time point. A representative flow cytometry analysis at P1 of MSC 6 cultured in the presence of PI-PL2 is shown in Figure 9 (a-d). Table 3 gives in detail the expression of constitutive antigens of MSC, in head to head experiments between different lots of PL and PI-PL, tested on MSC1, MSC3, MSC4, MSC5, MSC6, MSC7 and MSC8.

**Table 3:** Antigenic analysis by flow cytometry of MSC expanded with control PL and PI-PL preparations from P1 to P3

**MSC**	**PL or PI-PL**	**CD105+%**		**CD90+%**		**CD71+%**		**CD73+%**	
		**P1**^**@**^	P3*	**P1**^**§**^	**P3**^**#**^	**P1**^**+**^	**P3**^**^**^	**P1°**	**P3_**
**MSC-1**	**PL1**	92	98	92	98	95	99	95	99
	**PI-PL1**	94	99	92	99	95	100	95	100
**MSC-3**	**PL1**	93	98	94	98	95	99	95	99
	**PI-PL2**	93	95	90	90	95	98	95	98
**MSC-6**	**PL1**	94	96	93	99	94	99	94	99
	**PI-PL3**	91	98	94	98	95	99	95	99
**MSC-3**	**PL2**	94	97	91	97	92	98	92	98
	**PI-PL1**	94	97	95	98	95	99	95	99
**MSC-4**	**PL2**	93	96	92	98	94	99	90	99
	**PI-PL2**	93	98	92	97	94	99	94	99
**MSC-7**	**PL2**	93	96	91	99	92	98	95	99
	**PI-PL3**	92	96	94	98	95	98	95	98
**MSC-5**	**PL3**	90	96	90	98	94	99	94	99
	**PI-PL1**	93	98	92	98	94	99	94	99
**MSC-6**	**PL3**	93	97	95	99	90	98	92	99
	**PI-PL2**	92	98	94	99	94	99	94	99
**MSC-8**	**PL3**	92	99	94	98	95	99	93	96
	**PI-PL 3**	90	99	92	97	94	99	90	98

*PL* platelet lysate, *PI-PL* pathogen inactivated platelet lysate, *MSC* mesenchymal stem cells, *P1* passage 1 of culture, *P3* passage 3 of culture; CD34 and CD45 expression was consitently lower than 5% in all cultures at any time point. Each antigen is expressed as percentage of positive cells detected at P1 and P3 in each flow cytometric analysis performed on different MSC populations (1,3,4,5,6,7,8) which were grown in the presence of the indicated PL or PI-PL. ^@^p = 0.833, *p = 0.402, ^§^p = 0.615, ^#^p = 0.254, ^**+**^p = 0.095, ^^^p = 0.346, °p = 0.287 and _p = 0.512 at paired two-tailed Student’s T test between antigen expression in cultures supported by control PL and PI-PL lots.

**Figure 10:**
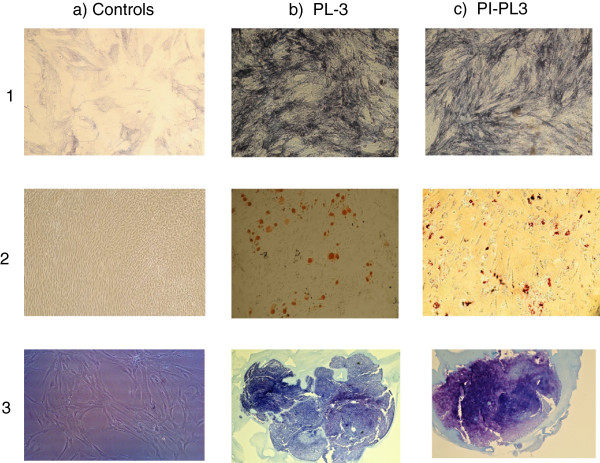
**MSC differentiation.** Multilineage differentiation documented by specific tissue staining and by microscope examination after differentiation of MSC 6 pre-expanded in the presence of PL3 (b) and PI-PL3 (c); **a**-(1-3) pictures are controls of cell staining in the absence of differentiating stimulus; **b**-(1-3) pictures represent osteoblast, adypocyte and chondrocyte differentiation of PL3-derived MSC6; **c**-(1-3) pictures represent osteoblast, adypocyte and chondrocyte differentiation of PI-PL3-derived MSC6.

As to the MSC multilineage differentiation potential, our results demonstrate that fresh or thawed MSC cultured in proper media, additioned with PL or PI-PL lots, were able to initiate differentiation into osteoblasts, adipocytes and chondrocytes. Figure 10 (a) 1-3 staining controls of undifferentiated MSC; b)1, c)1 osteogenesis; b)2, c)2 adipogenesis; b)3, c)3 chondrogenesis] shows the results of a representative experiment obtained by inducing differentiation of MSC 6 pre-expanded in the presence of PL3 or PI-PL3. Differentiation analysis was performed by specific tissue staining and microscope examination.

### MSC immunoregulatory effect

To test whether PI procedure could affect the immunosuppressive activity of MSC, one-way MLC and PHA induced proliferation assays were established. Figure 2 (a) shows a typical CFSE dilution profile following a proliferation stimulus in MLC, in the absence of any inhibitory effect; in the presence of MSC, most of proliferating cells fall into the first generation peak and do not move to the second one (b,c), as result of MSC immunosuppressive action. A proliferative inhibition ranging between 13% and 75% was found both in culture with PL or PI-PL derived MSC and the higher suppression of cell proliferation was found when 50,000 MSC were added to the culture, corresponding to a ratio of proliferating cells to MSC of 20/1 (Figure 2d). The inhibition of cell proliferation induced by PHA, in the presence of both PL-derived MSC and PI-PL-derived MSC (using a ratio of proliferating cells to MSC of 20/1 without stimulator cells) occurred to a less extent, ranging between 12%-25% (Figure 2d). Moreover, co-culture of PL-MSC or PI-PL-MSC with allogeneic PBMC highly increased the expression of CD25+ on CD4+ proliferating lymphocytes and induced the expansion of Treg Foxp3+ cells within CD25+ T subset, which are known to exhert immunosuppressive capacity. Figure 3 presents the dot plots of a representative experiment carried out on PBMC, cocultured with or without PI-PL3-derived MSC8, showing higher induction of CD4+/25+/FoxP3+ cells in the presence of MSC at a PBMC/MSC ratio of 20/1.

## Conclusions

The use of animal sera for clinical-scale MSC expansion raises concerns because of the risk to transmit prions, viruses or to induce immunological reactions in recipients following infusion and for these reasons these products are not allowed in good manufacturing practice (GMP) applied to cell therapy. Therefore, culture supplements derived from humans have been investigated in order to replace animal serum in MSC cultures. Human AB blood serum and umbilical cord blood serum have been found to provide a stimulus for MSC growth comparable to animal serum or higher with the preservation of MSC capacity to differentiate in three different mesodermal lineages [21]. Human PL is currently under investigation for its ability to sustain *ex vivo* MSC growth and data have been reported comparing the efficacy of PL with animal serum in promoting MSC expansion in the setting of cell-therapies [14-16]. From these evidences appear that PL is a powerful substitute of animal serum and its use may contribute to ameliorate the safety of MSC-based products in terms of immunological side effects and xenogenic pathogen transmission. PL properties are based on the release of growth factors by PLT which play a physiological role in the wound healing process. The content of PDGF-AB, TGF-β1 and bFGF in PL may account for its efficacy in promoting MSC growth since these cells have been shown to express surface receptors for PLT derived factors [14]. Nevertheless, procedures to prepare PL are highly variable and although this human source is rigorously screened for potential infectious threats, the risk of disease transmission by human derived products still requires careful consideration. In this study, a standardized procedure to generate PL by pooling multiple units of BC-derived PLT is described. The protocol includes a step of PI by Intercept technology, which is an approved and routinely applied practice for production of PLT concentrates for transfusional use in European countries [22], and the final resuspension of the PLT product in AS with a minimum residue of plasma (20-30%), as compared to the original products. PI technology by exposure to psoralen compound and UVA light has been found effective in inactivating a broad panel of viruses and bacteria without compromising PLT hemostatic function [23] and, therefore, PI-PL may represent a safer product for *ex vivo* MSC expansion to generate clinical grade cells for therapeutic strategies. However, a relevant issue is that to investigate whether pathogen reduction step may affect PL in its ability to generate *ex vivo* MSC. Here, the results of growth, expansion, differentiation and immunosuppressive function of several MSC populations cultivated in medium supplemented with both PL and PI-PL showed comparable features and growth potential. Differences between the various preparations, even though not relevant, may depends on the individual variability in PLT growth factor content. To obtain a more standardized PL preparation, we generated PI-PL by a pooling procedure which combined 12 PLT units, starting from 2 initial pools of 6, being six the maximum number of units which may be pooled for the PI step by the Intercept technology. An additional advantage of PI-PL preparation is the very low amount of residual plasma which may prevent the occurrence of immunological adverse reactions, often related to the presence of this human component. Some authors produced a plasma-free PL in human albumin solution, called PL-HA, for *in vitro* expansion of MSC [24,25]. The authors showed that MSC expanded in medium supplemented with PL-HA proliferated more extensively with respect to cultures additioned with FBS or conventional PL. In the same context, Burnouf *et al.* and Shih *et al*. [26,27] developed a virally inactivated PL for cell cultures by a solvent detergent procedure for viral inactivation, followed by oil extraction and adsorption with activated charcoal or soybean oil extraction and centrifugation, respectively. These preparations had been tested in growing cell lines, as MG63 and SIRC, and for selection and expansion of adipose tissue MSC. Neverthless, these studies reported that PDGF-AB and VEGF were completely removed by the adsorption step with charcoal as well as bFGF was partially removed by soybean oil extraction; from this evidence we may argue that these products are not completely suitable for MSC growth due to the well-described action of these growth factors in supporting optimal MSC expansion.

In a recent work Fekete *et al*. [28] produced PL from pool of 4 PLT units irradiated and quarantined for at least 4 months. The authors released the PLT units for PL production only if the all four donors contributing to a PLT pool had tested again as negative for conventional transfusion-transmitted infectious diseases. This approach to guarantee a safer clinical grade PL requires a longer storage of the starting material before PL preparation and is depending on the donor comeback for control testing. The addition of PI may further contribute to the safety of PL preparation by covering also the potential risk of new emerging infectious agents. Actually, over recent years the threat of transfusion transmitted agents focuses the attention on emerging pathogens which can be present in the blood and may concern public health, as the case of West Nile Virus [29]. Testing donation for evidence of a new pathogen may be adopted when the test become available and is validated for donor screening. Therefore, pathogen reduction could be considered the prompt approach to struggle with emerging infection transmissible via blood. Cell amplification *by ex-vivo* cultures, in the presence of human or animal blood components, is not a process free from the potential risk of transmission of infectious disease or immunological reactions; hence, the safety of this procedure may be greatly improved by the use of a human plasma-poor standardized product, contained in lots with a known growth factor content and subjected to an effective PI process. In this setting, PI-PL preparations represent a safe plasma-poor, well-standardized platelet lysates, prepared in lots which may be characterized for growth factor content and specific functional activity; these preparations had been improved in their quality grade by adding PI step and, for these reasons, appear as ideal supplements to develop uniform and well standardized protocols for *ex vivo* expansion of MSC without affecting antigenic and functional characteristics of these cells, as may happen with some serum-free commercial media [30].

## Abbreviations

MSC: Mesenchymal stem cells; PL: Platelet lysate; PLT: Platelet; AS: Additive solution; PI: Pathogen inactivation; PI-PL: Pathogen inactivated platelet lysate; BM: Bone marrow; BC: Buffy-coats; CAD: Compound absorption device; FBS: Fetal bovine serum; MNC: Mononuclear cells; PBMC: Peripheral blood mononuclear cells; PDGF-AB: Platelet derived growth factor AB; VEGF: Vascular endothelial growth factor; bFGF: Basic fibroblast growth factor; TGF-beta1: Transforming growth factor-beta1; DMSO: Dymethyl-sulfoxide; MLC: Mixed Lymphocyte Culture; PHA: Phytohaemmaglutinin; CFSE: CarboxyFluorescein di-acetate succinimidyl ester; CFU-F: Colony forming unit-fibroblast; ELISA: Enzyme-linked immunoSorbent assay; DT: Doubling time.

## Competing interests

The authors declare that they have no competing interests.

## Authors’ contributions

PI, DF, GB, CL and PT conceived of and carried out experiments, analysed and interpreted data and drafted the manuscript. MM purchased PLT pools and PLT pathogen inactivated pools. LP, LF and MN contributed to study design, statistical analyses and manuscript revision. All authors read and approved the final manuscript.
